# Utilizing Clinical Transformation Criteria for Prognostic Stratification in Follicular Lymphoma Prior to Initial Immunochemotherapy

**DOI:** 10.3390/hematolrep16040060

**Published:** 2024-10-04

**Authors:** Yoshikazu Hori, Hiroki Hosoi, Takayuki Hiroi, Ke Wan, Shogo Murata, Masaya Morimoto, Toshiki Mushino, Akinori Nishikawa, Takashi Sonoki

**Affiliations:** 1 Department of Hematology/Oncology, Wakayama Medical University, Wakayama 641-8509, Japan; hori-y@wakayama-med.ac.jp (Y.H.); shogo@wakayama-med.ac.jp (S.M.); mushino@wakayama-med.ac.jp (T.M.); nishikaw@wakayama-med.ac.jp (A.N.); sonoki@wakayama-med.ac.jp (T.S.); 2 Department of Hematology, Kinan Hospital, Wakayama 646-8588, Japan; masamor@wakayama-med.ac.jp; 3 Department of Transfusion Medicine, Wakayama Medical University Hospital, Wakayama 641-8510, Japan; 4 Department of Internal Medicine, Kainan Municipal Medical Center, Wakayama 642-0002, Japan; 5 Clinical Study Support Center, Wakayama Medical University, Wakayama 641-8509, Japan; 6 Department of Infection Prevention and Control, Wakayama Medical University, Wakayama 641-8509, Japan; 7 Division of Medical Information, Wakayama Medical University Hospital, Wakayama 641-8510, Japan

**Keywords:** follicular lymphoma, transformation, early progression, prognostic factor

## Abstract

Background: Although the prognosis of follicular lymphoma (FL) has improved, some patients experience early disease progression, including progression of disease within 24 months (POD24). Histological transformation is a critical event in FL. However, the heterogeneity of FL tumors makes it challenging to diagnose transformation accurately. We retrospectively applied the clinical transformation criteria used for FL transformation assessments at relapse or disease progression to conduct transformation assessments before the initial immunochemotherapy. Methods: Sixty-six FL patients who first received immunochemotherapy between January 2009 and February 2023 at our institution were selected. Twenty-three were clinical-transformation-positive (CLT+). Results: The progression-free survival (PFS) rate of the CLT+ patients was significantly lower than that of the clinical-transformation-negative (CLT−) patients. In the POD24 assessment subgroup, the CLT+ patients had a higher incidence of POD24 than the CLT− patients. There was no significant difference in PFS between the patients treated with CHOP-like regimens and those treated with bendamustine regimens. In the CHOP-like group, the CLT+ patients exhibited significantly lower PFS than the CLT− patients. In the bendamustine group, the clinical transformation did not affect PFS. Conclusion: Clinical transformation criteria may be useful for the prognostic stratification of FL prior to immunochemotherapy. Additionally, they may serve as predictors of POD24.

## 1. Introduction

Follicular lymphoma (FL) is the second most common type of B-cell lymphoma [1,2]. FL is a low-grade B-cell lymphoma with a long natural history and favorable outcomes [3]. In addition, the introduction of anti-CD20 antibodies as first-line therapies has improved its overall survival (OS) time to more than 15 years [4]. On the other hand, some patients with FL experience early relapse. Prognostic prediction plays a crucial role in determining the optimal treatment plan for FL. Various prognostic scoring systems for FL, including the Follicular Lymphoma International Prognostic Index (FLIPI), have been proposed [5].

The histological transformation of FL into diffuse large B-cell lymphoma (DLBCL) is a critical event in the clinical course of FL [6]. Transformation leads to the rapid progression of the disease, i.e., it becomes a form of aggressive lymphoma. Transformation is defined by the histological documentation of large cells that eradicate the follicular architecture [6]. Therefore, a biopsy is necessary to confirm the diagnosis. However, biopsies may not capture all transformed lesions because FL exhibits tumor heterogeneity [7]. In FL patients that exhibit transformation at relapse or progression, clinical features such as rapid lymph node enlargement and sudden increases in lactate dehydrogenase (LDH) levels are commonly observed [2]. However, it remains unclear whether clinical features suggestive of transformation are also associated with the prognosis of FL prior to the initial treatment. Therefore, we investigated the significance of clinical features that suggest transformation prior to the initial treatment. Specifically, we examined whether findings previously reported to be useful for clinically diagnosing transformation at the time of disease progression or relapse are also useful for such purposes before starting treatment. Then, we examined whether the presence/absence of predictors of clinical transformation prior to the initial treatment has a prognostic impact.

## 2. Materials and Methods

### 2.1. Patients and Study Design

This was a single-center, retrospective study. Patients over the age of 20 years, who had been diagnosed with grade 1–3a FL and who underwent their initial immunochemotherapy at Wakayama Medical University Hospital between January 2009 and February 2023, were selected via a review of electronic medical records. Patients with FL grade 3b or those diagnosed with other concomitant lymphomas, such as composite lymphoma involving FL and DLBCL, were excluded. The initial immunochemotherapy consisted of CHOP (cyclophosphamide, doxorubicin, vincristine, and prednisolone)-like therapy or bendamustine therapy, plus an anti-CD20 antibody (rituximab or obinutuzumab) [8,9]. The CHOP-like regimens included a THP-COP (pirarubicin, cyclophosphamide, vincristine, and prednisone) regimen in addition to the CHOP regimen [10,11]. Patients who received other chemotherapy regimens were excluded. Patients who received rituximab monotherapy or radiotherapy prior to the initial immunochemotherapy were included in the study. The date of the initial immunochemotherapy was defined as the starting point for the observation period. Maintenance therapy with rituximab or obinutuzumab was administered at the discretion of the attending physician. This study was approved by the ethics committee of Wakayama Medical University (Approval No. 3833, 17 June 2024).

### 2.2. Definitions

We modified previously reported clinical transformation criteria, which were intended to be used to diagnose clinical transformation at relapse or disease progression, so that they could be used to diagnose clinical transformation at the initial presentation. The previously reported clinical transformation criteria were based on the presence of at least one of the following: a sudden rise in the LDH level to more than twice the upper limit of normal, rapid discordant localized nodal growth, new involvement at unusual extranodal sites (e.g., the liver, bone, muscle, or brain), new B symptoms, or the development of new hypercalcemia [12]. In our study, we modified the previously reported criteria to be applicable at the initial immunochemotherapy and defined clinical transformation when any of the following findings were met: an increase in the LDH level to more than twice the reference value, hypercalcemia, rapid lymph node enlargement, or the appearance of atypical extranodal lesions. Rapid lymph node enlargement was defined as a more than two-fold increase in the sum of the products of the longest perpendicular diameters within the three months before the start of immunochemotherapy. Atypical extranodal lesions were defined as lesions affecting the bone/bone marrow, liver, muscle, lung, pleura, skin, brain, or nerves, as previously reported [12]. The sites of lesions were assessed using Positron emission tomography-computed tomography (PET/CT) imaging. The equipment used was either the Philips Ingenuity TF or Philips GEMINI TF (Philips Healthcare, Best, The Netherlands). Our study classified cases demonstrating significant fluorodeoxyglucose (FDG) uptake in the bone marrow on PET/CT imaging as clinical-transformation-positive. In contrast, cases that only showed bone marrow infiltration in bone marrow biopsies were not classified as clinical-transformation-positive. Since PET/CT cannot strictly differentiate between FDG uptake by bone and FDG uptake by the bone marrow, uptake by either the bone or bone marrow was considered equivalent for the analysis based on the previously reported classification and study [13,14]. The identification of relapse relied on assessments performed by radiologists, with the date of relapse defined as the day of the acquisition of the image indicating relapse. CT or PET/CT imaging was conducted every 6 to 12 months after the completion of treatment at the attending physician’s discretion. Patients who relapsed or exhibited disease progression within 24 months (POD24) of the initiation of immunochemotherapy were defined as POD24-positive [15]. In the analysis of POD24, patients with a follow-up period of less than 24 months after the initial immunochemotherapy were excluded in accordance with previous studies on POD24 [15,16].

### 2.3. Statistical Analyses

Electronic and paper medical records were used to review the patients’ medical histories. All characteristic-related data were summarized using Microsoft Excel 365 (Microsoft, Redmond, WA, USA). Comparisons of categorical variables were conducted using Fisher’s exact test. The Mann–Whitney *U*-test was used to analyze continuous variables. The primary endpoint was progression-free survival (PFS). PFS was calculated from the initial immunochemotherapy to disease progression, relapse, or the last follow-up. The secondary endpoint was OS. OS was calculated from the initial immunochemotherapy until death from any cause or the last follow-up. Data were censored at the date of the last follow-up, at which the patient’s survival was checked on 31 May 2024. PFS and OS were estimated using the Kaplan–Meier method and were compared using the log-rank test. The Cox proportional hazards regression model was used in the univariate analyses of PFS. *p*-values of < 0.05 were considered significant. Statistical analyses were performed using the software EZR version 1.55 (Saitama Medical Center, Saitama, Japan) [17].

## 3. Results

### 3.1. Patient Characteristics

Sixty-six FL patients were included in this study. Their median age was 65 years (range 33–86). Histological examinations revealed that 15 (23%), 26 (39%), and 19 (29%) patients had grade 1, 2, and 3a histology, respectively (Table 1). Thirty-five patients (53%) had high-risk FLIPI scores. Fifty-six patients (85%) had Ann Arbor stage III or IV disease. Bone marrow involvement was seen in 28 patients (42%) in the bone marrow biopsy assessments. Thirty patients (45%) received a CHOP-like regimen. Maintenance therapy was administered to 23 patients (35%) after the initial immunochemotherapy. No patients received autologous hematopoietic stem cell transplantation (ASCT) following the initial chemotherapy, whereas ASCT was performed in four patients after chemotherapy for relapse.

In comparing the characteristics of patients in the clinical-transformation-negative and -positive groups, there was a tendency for the clinical-transformation-positive group to have a higher incidence of elevated LDH levels before starting immunochemotherapy, although this difference did not reach statistical significance (*p* = 0.0631). In addition, the clinical-transformation-positive group included a significantly higher proportion of patients who received rituximab as a monoclonal antibody (*p* = 0.00785). There was no significant difference in the proportion of patients receiving maintenance therapy with anti-CD20 antibodies after the initial immunochemotherapy between the two groups (*p* = 0.115). No significant differences were observed between the two groups in terms of other factors.

In the patients that received CHOP-like regimens, the concomitant use of anti-CD20 antibodies involved the use of rituximab in 28 patients (93%) and obinutuzumab in 2 patients (7%). On the other hand, among the patients treated with the bendamustine regimen, the combined use of rituximab or obinutuzumab was observed in 11 (31%) and 25 (69%) patients, respectively (Supplementary Table S1). The combined use of obinutuzumab was significantly more common among the patients who received the bendamustine regimen than among those who received CHOP-like regimens (*p* < 0.001). After the availability of bendamustine for the initial treatment of FL in Japan, 36 patients received the bendamustine regimen, and ten patients received CHOP-like regimens.

### 3.2. The Factors Related to Clinical Transformation

The factors that resulted in positivity for clinical transformation in the 23 patients are shown in Table 2. The presence of atypical extranodal lesions was the most common factor associated with clinical transformation, being observed in 18 patients (78%). Extranodal lesions predominantly arose in the bone or bone marrow, accounting for 13 patients (57%). Among the 22 patients who experienced relapse or progression, a rebiopsy was conducted in 10 patients (45%). Among these 10 cases, clinical transformation was positive at the initial diagnosis in 6 patients. Three patients with clinical transformation were diagnosed with DLBCL based on rebiopsies.

### 3.3. Stratification of PFS according to the FLIPI or the Presence/Absence of Clinical Transformation

**Figure 1:**
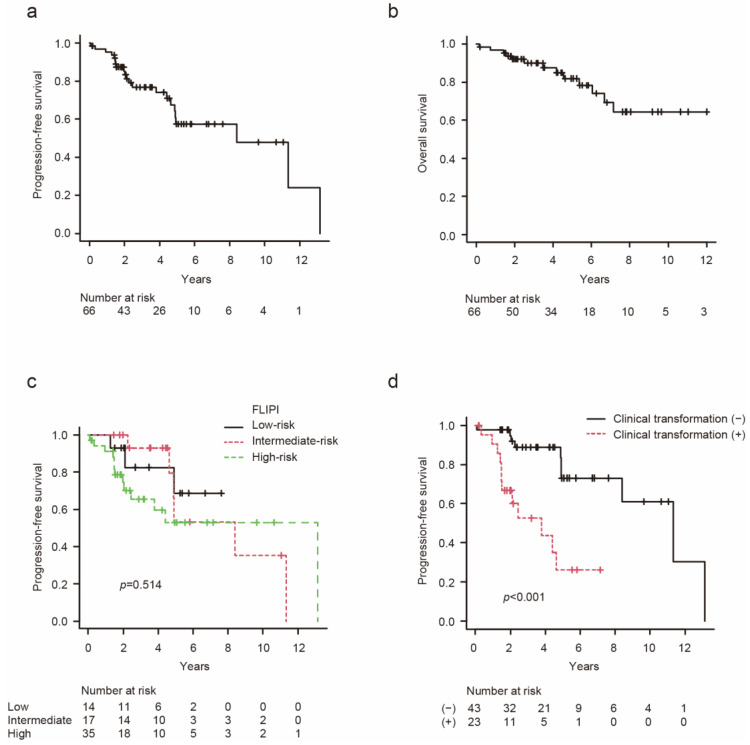
Survival curves: Progression-free survival (**a**) and overall survival (**b**) for all patients. Progression-free survival according to the Follicular Lymphoma International Prognostic Index (FLIPI) (**c**) and the clinical transformation criteria met before the initial immunochemotherapy (**d**). Progression-free survival and overall survival were calculated from the initial immunochemotherapy.

The median duration of the follow-up period was 4.2 years (range: 0.17–16.8) among all patients. There was no significant difference in the duration of the follow-up period between the clinical-transformation-negative and -positive groups (median: 4.2 years (range: 1.4–16.8) vs. 3.5 (range: 0.2–8.0), *p* = 0.238). The 3-year and 5-year PFS rates for all patients were 76.9% (95% confidence interval [CI]: 63.2–86.0) and 57.4% (95% CI: 40.1–71.4), respectively (Figure 1a). The 3-year and 5-year OS rates for all patients were 90.0% (95% CI: 78.9–95.4) and 81.9% (95% CI: 67.3–90.4), respectively (Figure 1b).

PFS was assessed according to the FLIPI classification and the presence/absence of clinical transformation. The 3-year PFS rates for the FLIPI low-risk, intermediate-risk, and high-risk groups were 82.5% (95% CI: 45.1–95.5), 92.9% (95% CI: 59.1–99.0), and 65.6% (95% CI: 44.7–80.2), respectively (Figure 1c). In our cohort, there was no significant difference in PFS according to the FLIPI classification (*p* = 0.514). In contrast, the PFS rate of the clinical-transformation-positive patients was significantly lower than that of the clinical-transformation-negative patients (3-year PFS: 52.5% (95% CI: 27.4–72.6) vs. 88.7% (95% CI: 72.5–95.6), *p* < 0.001, Figure 1d). The clinical transformation positivity was related to significantly inferior PFS (hazard ratio [HR]: 5.32, 95% CI: 2.07–13.7, *p* < 0.001). The rapid increase in LDH and the appearance of atypical extranodal lesions were each associated with poor PFS, with HR of 4.88 (95% CI: 1.04–22.9, *p* = 0.044) and 4.96 (95% CI: 1.04–22.9, *p* < 0.0001), respectively.

### 3.4. The Impact of Clinical Transformation on PFS according to the Administered Regimen

**Figure 2:**
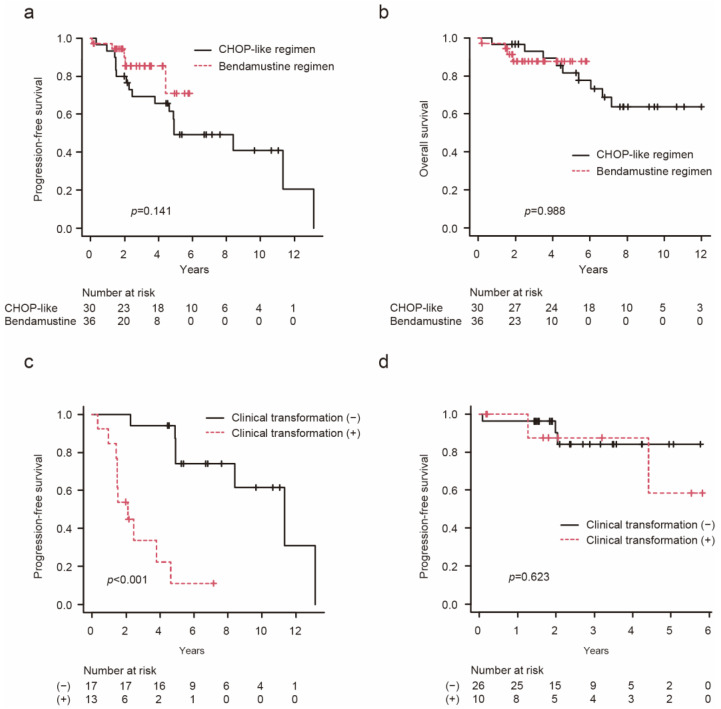
Survival curves according to the chemotherapy regimen: Progression-free survival (**a**) and overall survival (**b**) according to the chemotherapy regimen. Progression-free survival in patients treated with a CHOP-like regimen (**c**) or the bendamustine regimen (**d**) according to the clinical transformation criteria met before the initial immunochemotherapy. Progression-free survival and overall survival were calculated from the initial immunochemotherapy. CHOP, a combination of cyclophosphamide, doxorubicin, vincristine, and prednisolone.

PFS was evaluated separately in the patients who received CHOP-like regimens and those who received the bendamustine regimen. The duration of the follow-up period among the patients treated with the bendamustine regimen was significantly shorter than that among the patients treated with a CHOP-like regimen (*p* < 0.001). There was no difference in PFS between these groups (*p* = 0.141, Figure 2a) in addition to OS (*p* = 0.988, Figure 2b). Among the patients who received CHOP-like regimens, those with clinical transformation showed significantly lower PFS than those who were clinical-transformation-negative (*p* < 0.001, Figure 2c). Conversely, among the patients who received bendamustine therapy, no significant difference in PFS was observed between the clinical-transformation-positive and -negative patients (*p* = 0.623, Figure 2d).

### 3.5. The Impact of Clinical Transformation on POD24

**Figure 3:**
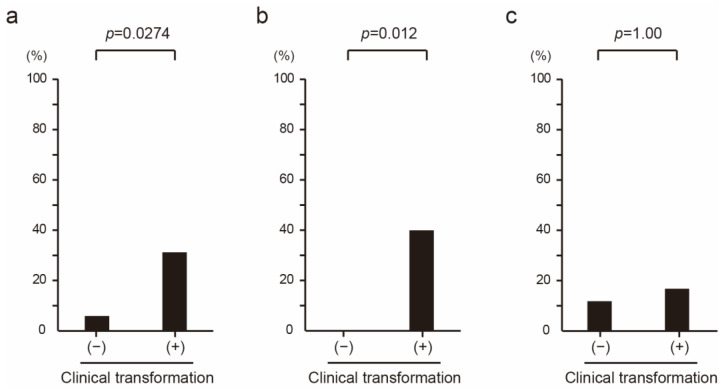
The proportion of patients with POD24 according to whether they met the criteria for clinical transformation: (**a**) All patients (*n* = 50). (**b**) Patients treated with CHOP-like regimens (*n* = 27). (**c**) Patients treated with bendamustine regimen (*n* = 23).

POD24 could be evaluated in 50 patients who have at least 24 months of follow-up. Of the patients in whom POD24 was evaluable, 16 (32%) were clinical-transformation-positive. Of these, two clinical-transformation-negative patients (6%) and five clinical-transformation-positive patients (31%) exhibited POD24 (Figure 3a). The proportion of patients that demonstrated POD24 was significantly higher among the clinical-transformation-positive patients than among the clinical-transformation-negative patients (*p* = 0.0274). Of the 50 patients evaluated for POD24, 27 received a CHOP-based regimen, and 23 received the bendamustine regimen. Among the patients that received CHOP-like regimens, 0 (0%) of the 17 clinical-transformation-negative patients and 4 (40%) of the 10 clinical-transformation-positive patients exhibited POD24 (*p* = 0.012, Figure 3b). On the other hand, among the patients who received the bendamustine regimen, 2 (12%) of the 17 clinical-transformation-negative patients and 1 (17%) of the 6 clinical-transformation-positive patients exhibited POD24 (*p* = 1.00, Figure 3c).

## 4. Discussion

We retrospectively examined the prognosis of FL patients after their initial treatment, focusing on clinical findings indicative of transformation. The clinical-transformation-positive patients exhibited significantly lower PFS than those who were clinical-transformation-negative. Among the patients in whom POD24 was evaluated, the clinical-transformation-positive patients had a higher incidence of POD24 than the clinical-transformation-negative patients. Although among the patients that received CHOP-like regimens, the clinical-transformation-positive patients showed lower PFS than the clinical-transformation-negative patients, no significant difference in PFS was observed between these groups among the patients that received the bendamustine regimen.

Prognostic factors for FL based on the initial clinical findings, such as the FLIPI, FLIPI2, and more simple indices, have been examined before and after the rituximab era [2,5,18,19,20]. Our study assessed atypical extranodal involvement, which has not often been considered a prognostic factor for FL, at the initial presentation. While previous studies regarded bone marrow involvement at the initial diagnosis as a risk factor for prognosis, we classified bone marrow involvement indicated by abnormal FDG accumulation on PET/CT as “involvement at an atypical extranodal site” and considered such cases to be positive for clinical transformation. Accumulation of FDG in bone marrow on PET/CT at initial diagnosis reflects bone marrow involvement and has been reported to be associated with prognosis in FL [14,21,22,23]. Recently, genetic mutations have been reported to be useful for prognostic stratification in FL [24,25]. However, there are issues regarding the cost of testing for genetic mutations in routine clinical practice, which were not addressed in our study. The factors examined in our study were based on those used to diagnose clinical transformation at recurrence in a previous study and enable prognostic stratification after the initial treatment through an easy assessment that could be employed in clinical practice [15].

Transformation is seen at relapse or progression in 2–3% of FL patients per year and is associated with rapid progression, early progression, treatment resistance, and a worse prognosis [6,12,26,27,28,29,30,31]. On the other hand, when a transformation is histologically confirmed at the initial presentation, the diagnosis shifts from FL to a more aggressive form of lymphoma, such as DLBCL. FL tumors exhibit spatial heterogeneity [7]. Therefore, even if biopsies of easily accessible sites do not show histological transformation, lesions at other sites may have undergone transformation. Similarly, at the relapse of FL, the presence/absence of transformation may be determined differently depending on the biopsy site. When transformation is clinically suspected due to relapse, clinically diagnosing transformation has been reported to be useful [12]. Worse prognoses were reported in cases of FL involving transformation, including those in which transformation was diagnosed clinically without histological confirmation [12,32,33]. Since, due to tumor heterogeneity, there are sites other than biopsy sites at which transformation may or is likely to occur, even before the initial treatment, we adapted the clinical transformation criteria so that they were appropriate for prognostic prediction before the initial treatment. Our results show that factors suggestive of clinical transformation could be used for prognostic stratification before the initial chemotherapy.

The early recurrence of FL after the initial immunochemotherapy occurs in 10–20% of patients [15,34]. Early relapse has been broadly defined as a recurrence that arises within two years of frontline immunochemotherapy or two years of diagnosis, which is also referred to as POD24 [35]. POD24 has been reported to be a robust prognostic factor for assessing OS in patients receiving R-CHOP treatment [15,36,37]. In addition, POD24 was also found to be related to worse OS in patients receiving a bendamustine–rituximab regimen [38]. The latter study showed that the majority of patients who experienced POD24 had transformed disease and demonstrated that the presence of occult or early transformation is the main driver of POD24. Some studies found that transformation impacted survival more than POD24 [16,39]. In our study, patients who were positive for clinical transformation developed POD24 more often than those who were clinical-transformation-negative. Our findings also suggest that the clinical transformation criteria employed in this study are associated with POD24. The presence/absence of POD24 is determined by observing post-treatment progress; hence, it is limited because it is not applicable to prognostication before the start of chemotherapy. In contrast, the method used to assess clinical transformation in our study has the advantage of being applicable to prognostic prediction prior to the initial treatment and may also be useful for predicting POD24.

In our study, clinical transformation did not have any apparent effect on PFS in the patients treated with the bendamustine regimen. In the bendamustine regimen group, PFS may have been influenced by the higher proportion of patients receiving obinutuzumab instead of rituximab. Obinutuzumab is a novel anti-CD20 antibody, which has been modified to give it greater antibody-dependent cell-mediated cytotoxicity than rituximab. Although previous studies have demonstrated that there was no significant difference in the incidence of transformation between patients treated with combination therapy involving rituximab and those treated with combination therapy involving obinutuzumab, further studies are warranted to investigate the impact of clinical transformation criteria in patients treated with obinutuzumab [9,40].

This study had several limitations. First, it was a single-center retrospective study with a small sample size. Thus, the attending physicians’ opinions may have influenced the choice of treatment regimen; however, decisions regarding the treatment strategy were made during conferences. Second, the timing of the initial treatment affected which anticancer agents were available through the Japanese national health insurance system. As bendamustine was available for the initial treatment of FL in December 2016, patients who underwent their initial immunochemotherapy between January 2009 and November 2016 received a CHOP-like regimen. Obinutuzumab was launched in August 2018. Consequently, patients who received their initial immunochemotherapy before August 2018 were administered rituximab. Third, we were unable to investigate tumor-specific biological factors such as genetic mutations. Recently, genetic abnormalities, as incorporated in tools like m7-FLIPI, have been recognized as risk factors for disease progression in FL [24]. However, during the study period, genetic testing had not yet become widely implemented in routine clinical practice, and thus, we did not have access to genetic data [41]. On the other hand, genetic abnormalities detected in tissue samples may not fully reflect the tumor heterogeneity of FL and may not accurately determine transformation. Detecting genetic abnormalities using cell-free DNA derived from plasma may better capture tumor heterogeneity in the future. However, we believe that prognostic stratification based on clinical data, as used in this study, still holds an important role in current clinical practice [42].

In conclusion, the presence/absence of clinical transformation was suggested to be useful for the prognostic stratification of FL patients before the start of immunochemotherapy. While the association between clinical transformation and histological transformation needs to be evaluated, the tumor heterogeneity seen in FL complicates the accurate assessment of transformation. In the future, it is anticipated that novel techniques, such as examinations of cell-free DNA, will be useful for evaluating tumor heterogeneity.

## Figures and Tables

**Table 1:** Patient characteristics according to presence/absence of clinical transformation.

Characteristics	Total (*n* = 66)	Clinical Transformation (−) (*n* = 43)	Clinical Transformation (+) (*n* = 23)	*p*-Value
Age, median (range)	65 (33–86)	64 (33–79)	64 (42–86)	0.167
Male, n (%)	31 (47)	21 (49)	10 (43)	0.797
ECOG PS 0–1, n (%)	65 (98)	43 (100)	22 (96)	0.348
Follicular lymphoma histological grade, n (%)				
Grade 1	15 (23)	11 (26)	4 (17)	0.0932
Grade 2	26 (39)	18 (42)	8 (35)	
Grade 3a	19 (29)	13 (30)	6 (26)	
Missing data	6 (9)	1 (2)	5 (22)	
FLIPI score, n (%)				
Low (0–1)	14 (21)	11 (26)	3 (13)	0.173
Intermediate (2)	17 (26)	13 (30)	4 (17)	
High (3–5)	35 (53)	19 (44)	16 (70)	
Ann Arbor stage, n (%)				
I/II	10 (15)	9 (21)	1 (4)	0.146
III/IV	56 (85)	34 (79)	22 (96)	
Bone marrow involvement, n (%)				
Negative	35 (53)	25 (58)	10 (43)	0.305
Positive	28 (42)	17 (40)	11 (48)	
Missing	3 (5)	1 (2)	2 (9)	
Anemia (Hb < 12 g/dL), n (%)	14 (21)	7 (16)	7 (30)	0.215
Elevated LDH (>normal), n (%)	26 (39)	13 (30)	13 (57)	0.0631
Elevated sIL-2R (>496 U/mL), n (%)	61 (92)	38 (88)	23 (100)	0.154
β2-microglobulin value, n (%)				
≤2 mg/L	17 (26)	13 (30)	4 (17)	0.557
>2 mg/L	33 (50)	20 (47)	13 (57)	
Missing data	16 (24)	10 (23)	6 (26)	
Median SUVmax in PET/CT examination (range)	8.72 (3.48–26.43)	8.54 (3.48–26.43)	8.805 (4.49–20.89)	0.467
Missing, n (%)	7 (11)	4 (9)	3 (13)	0.687
Regimen				
CHOP-like	30 (45)	17 (40)	13 (57)	0.206
Bendamustine	36 (55)	26 (60)	10 (43)	
Monoclonal antibodies used for the first-line immunochemotherapy				
Rituximab	39 (59)	20 (47)	19 (83)	0.00785
Obinutuzumab	27 (41)	23 (53)	4 (17)	
Maintenance therapy after the initial immunochemotherapy				
Yes	23 (35)	18 (42)	5 (22)	0.115
No	43 (65)	25 (58)	18 (78)	

Bone marrow involvement was assessed by bone marrow biopsy. ECOG, Eastern Cooperative Oncology Group; PS, performance status; FLIPI, Follicular Lymphoma International Prognostic Index; Hb, hemoglobin; LDH, lactate dehydrogenase; sIL-2R, soluble interleukin-2 receptor; SUVmax, maximum standardized uptake value; PET/CT, positron emission tomography-computed tomography; CHOP, cyclophosphamide, doxorubicin, vincristine, and prednisolone.

**Table 2:** Factors associated with clinical transformation.

Factors (*n* = 23)	Clinical Transformation (+) *n* (%)
Rapid increase in LDH	4 (17%)
Hypercalcemia	0 (0%)
Rapid lymph node enlargement	2 (9%)
Appearance of atypical extranodal lesions	18 (78%)
Bone/bone marrow	13 (57%)
Lung	2 (9%)
Pleura	2 (9%)
Skin	1 (4%)
Nerves	1 (4%)

There was one case in which the two factors (increased LDH levels and extranodal lesions) were seen. LDH, lactate dehydrogenase.

## Data Availability

The dataset analyzed in this study is available from the corresponding author upon reasonable request.

## Supplementary Materials

The following supporting information can be downloaded at https://www.mdpi.com/article/10.3390/hematolrep16040060/s1, Table S1: Patient characteristics according to treatment regimen.
