# Supplementary material for: Utilizing Clinical Transformation Criteria for Prognostic Stratification in Follicular Lymphoma Prior to Initial Immunochemotherapy
Source: Hematol Rep. 2024 Oct 4;16(4):612–23. doi: 10.3390/hematolrep16040060 (PMC11503408; doi:10.3390/hematolrep16040060)
Supplement: Supplementary file 1 [file hematolrep-16-00060-s001.zip › hematolrep-3135901-supplementary.pdf]

Supplementary table S1

Patient characteristics according to treatment regimen

Characteristics	Total (N=66)	CHOP-like regimen (N=30)	Bendamustine based regimen (N=36)	*P*-value
Median age (range)	65 (33-86)	65 (33-86)	65.5 (46-80)	
Male, n (%)	31 (47)	16 (53)	15 (41)	0.458
ECOG PS 0–1, n (%)	65 (98)	30 (100)	35 (97)	1
Follicular histological grade, n (%)				
Grade 1	15 (23)	6 (20)	9 (25)	0.255
Grade 2	26 (39)	10 (33)	16 (44)	
Grade 3a	19 (29)	9 (30)	10 (27)	
Unknown	6 (9)	5 (16)	1 (2)	
FLIPI score, n (%)				
Low, 0–1	14 (21)	6 (20)	8 (22)	0.456
Intermediate, 2	17 (26)	10 (33)	7 (19)	
High, 3–5	35 (53)	14 (46)	21 (58)	
Clinical transformation positivity, n (%)	23 (34)	13 (43)	10 (27)	0.206
Ann Arbor stage, n (%)				
I/II	10 (15)	6 (20)	4 (11)	0.492
III/IV	56 (85)	24 (80)	32 (88)	
Bone marrow involvement, n (%)				
Negative	35 (53)	15 (50)	20 (55)	0.211
Positive	28 (42)	12 (40)	16 (44)	
Missing	3 (4)	3 (10)	0 (0)	
Anemia (Hb < 12 g/dL), n (%)	14 (21)	8 (26)	6 (16)	0.375
Elevated LDH (>normal)	26 (39)	11 (36)	15 (41)	0.802
Elevated sIL-2R (> 496 U/mL), n (%)	61 (92)	26 (86)	35 (97)	0.169
β2-microglobulin value, n (%)				
≤2 mg/L	17 (25)	6 (20)	11 (30)	0.025
>2 mg/L	33 (50)	12 (40)	21 (58)	
Missing	16 (24)	12 (40)	4 (11)	
PET examination, SUVmax (range)	8.72 (3.48-26.43)	8.43 (3.48-20.89)	8.81 (4.49-26.43)	
Missing	7 (11)	4 (13)	3 (8)	0.693
Monoclonal antibodies used for the first-line immunochemotherapy				
Rituximab	39 (59)	28 (93)	11 (31)	<0.001
Obinutuzumab	27 (41)	2 (7)	25 (69)	
Maintenance therapy after the initial immunochemotherapy				
Yes	23 (35)	9 (30)	14 (39)	0.605
No	43 (65)	21 (70)	22 (61)	

Bome marrow involvement was assessed by bone marrow biopsy.

CHOP, cyclophosphamide, doxorubicin, vincristine, and prednisolone

ECOG, Eastern Cooperative Oncology Group; PS, performance status; FLIPI, Follicular Lymphoma International Prognostic Index; LDH, lactate dehydrogenase; sIL-2R, soluble interleukin-2 receptor

SUVmax, maximum standardized uptake value; PET/CT, positron emission tomography-computed tomography
